# Broad-spectrum phage cocktail targeting *Campylobacter* improves survival in *Galleria mellonella*, a bridging host model for poultry biocontrol

**DOI:** 10.3389/fmicb.2026.1744469

**Published:** 2026-04-15

**Authors:** Estibaliz Ruiz-Santamaría, Amaia Lasagabaster, Gaizka Carregal, Katherine Miranda-Cadena, Estibaliz Mateo

**Affiliations:** 1 AZTI, Food Research, Basque Research and Technology Alliance (BRTA), Parque Tecnológico de Bizkaia, Derio, Spain; 2 Department of Immunology, Microbiology and Parasitology, Faculty of Medicine and Nursing, University of the Basque Country (UPV/EHU), Bilbao, Spain

**Keywords:** biocontrol, campylobacteriosis, *Galleria mellonella*, infection model, phage-therapy, poultry

## Abstract

*Campylobacter jejuni* and *Campylobacter coli* are the main agents of campylobacteriosis, a globally prevalent foodborne illness predominantly linked to the consumption of contaminated poultry products. The increasing antimicrobial resistance in *Campylobacter* requires innovative control strategies throughout the poultry production chain. Bacteriophages, highly specific bacterial viruses, represent a promising biocontrol approach capable of selectively targeting *Campylobacter* without disrupting the natural microbiota. However, early-stage validation in intermediate models, such as *Galleria mellonella*, is essential to ensure safety and efficacy before application in poultry, as has been established for other zoonotic pathogens. This study evaluated the *in vitro* and *in vivo* efficacy of a novel four-phage cocktail targeting *Campylobacter. In vitro* assays showed that the phage cocktail successfully lysed all 13 strains tested, and each individual phage displayed a broad lytic spectrum, with most strains being susceptible to multiple phages. *In vivo* virulence screening in *G. mellonella* revealed marked strain-dependent virulence, with only five of 13 strains reducing larval survival below 50%. Phage efficacy *in vivo* was optimized using *C. jejuni* CJE065, the most virulent strain in the model. The phage cocktail applied at MOI 10 increased the *G. mellonella* survival from 25.5% in untreated controls to 57.5% (*p* < 0.001), whereas lower MOIs provided only transient protection. Phage-antibiotic therapy combining phage cocktail and either erythromycin or ciprofloxacin further enhanced larval survival rates, reaching up to 88.8 and 83.8%, respectively (*p* < 0.001). Overall, these findings support the potential use of this phage cocktail as an early-stage intervention against *Campylobacter* and highlight *G. mellonella* as a suitable intermediate model for optimizing phage-based treatments while reducing the need for vertebrate models.

## 1 Introduction

*Campylobacter jejuni* and *Campylobacter coli* are the main causative species of campylobacteriosis, the most reported zoonosis in the European Union (EU) with more than 145,000 confirmed human cases in 2023 (European Food Safety Authority and European Centre for Disease Prevention and Control, 2024). This foodborne illness is prevalent worldwide and is often associated with the consumption of contaminated and undercooked meat, especially poultry.

In 2017, the World Health Organization (WHO) included *Campylobacter* in its priority list of antibiotic-resistant bacteria, although it was removed in 2024. However, both the European Food Safety Authority (EFSA) and the European Centre for Disease Prevention and Control (ECDC) continue to report an increasing trend in resistance to commonly used antimicrobials among *Campylobacter* isolates from humans and animals (Tacconelli et al., 2018; WHO, 2017, 2024; European Food Safety Authority and European Centre for Disease Prevention and Control, 2025). The remarkable ability of this pathogen to develop resistance to key antibiotics, such as fluoroquinolones (e.g., ciprofloxacin) and macrolides (e.g., erythromycin), compromises the effective treatment of campylobacteriosis. In addition, interspecies transfer of resistance further aggravates this public health problem (Woyda et al., 2023).

The prevention and control of *Campylobacter* at farm level constitutes a food safety issue, since it is a recognized risk factor for human campylobacteriosis. The prevalence of *Campylobacter* in poultry is predominantly caused by *C. jejuni* species followed by *C. coli* (Rossler et al., 2020). Although rarely detected in young birds, *Campylobacter* prevalence in broiler flocks increases with age, ranging from 47 to 75.8% at the flock-level and reaching up to 87.3% on carcasses at slaughter (Haems et al., 2024). Horizontal transmission of *Campylobacter* at farms is rapid and efficient, with most birds colonized within days, reaching levels between 10^6^ and 10^8^ colony forming units (CFU) per gram in their intestinal tract until slaughter (Marotta et al., 2015; Reichelt et al., 2022). The spread of *Campylobacter* during slaughter and subsequent processing of poultry food products may occur by cross-contamination of broiler carcasses by the intestinal content.

The high incidence of campylobacteriosis, the alarming emergence and dissemination of resistant *Campylobacter* strains, and its widespread prevalence on farms underscore the urgent need for innovative and effective control strategies.

Among these, the use of bacteriophages emerges as a promising alternative for the control of *Campylobacter* along the poultry chain. The high specificity of phages ensures the elimination of target pathogen without affecting the natural microbiota of the food, the host or the environment (Lavilla et al., 2023). *Campylobacter*-specific phages could be used as single agents or in combination as phage cocktails, to naturally reduce *Campylobacter* colonization in poultry and other animal reservoirs, with the aim of minimizing the incidence of human campylobacteriosis (Nafarrate et al., 2020; Olson et al., 2022; Lavilla et al., 2023).

The effectiveness of different phage treatments in reducing *Campylobacter,* including resistant strains, has been reported in previous *in vivo* trials involving broiler chickens (Carvalho et al., 2010; Mousavi et al., 2021; Lavilla et al., 2023; Peh et al., 2023; Peh et al., 2024), poultry products (Lavilla et al., 2023; Olson et al., 2022), and commercial farm environments (Chinivasagam et al., 2020). Moreover, the US Food and Drug Administration (FDA) approved in 2022 a commercially available phage-based product targeting *C. jejuni*, CampyShield™ (Intralytix, USA), which contains a cocktail of three to eight lytic phages and is intended for application on food surfaces (GRAS Notice No. GRN000966). CampyShield™ is authorized exclusively as a GRAS antimicrobial applied directly to raw meat and poultry surfaces and is not designed for *in vivo* therapeutic or preventive applications. Publicly available information indicates activity primarily against *C. jejuni* and some *C. coli* strains, without assessment across a broader diversity of *Campylobacter* species. Consequently, studies exploring phage efficacy against different *Campylobacter* species are needed, as well as the use of alternative *in vivo* models for evaluating the effectiveness of phages in monotherapy and in combination with antibiotics.

The screening and selection of the best phage candidates can initially be conducted using the invertebrate models such as *Galleria mellonella* as an alternative, thereby avoiding the ethical, economic, and methodological constraints associated with direct experimentation in broiler chickens (Carvalho et al., 2010; Mousavi et al., 2021; Kiani et al., 2022; Guo et al., 2024). Other invertebrate models, such as *Drosophila melanogaster* (Jang et al., 2019) or *Caenorhabditis elegans* (Fatima et al., 2025), have also been used to evaluate the efficacy of phages against human pathogens. In the case of *G. mellonella,* this infection model has previously been used to study the virulence of several foodborne pathogens, including *Campylobacter* (Senior et al., 2011; Bojanić et al., 2020; Luiz de Freitas et al., 2021; Vinueza-Burgos et al., 2023; Gomes et al., 2024; Yan et al., 2025), and has proven useful for optimizing antimicrobial interventions prior to validation in poultry (Lacharme-Lora et al., 2019; Nale et al., 2021; Ménard et al., 2021). However, although *G. mellonella* has been widely used to evaluate phage-based treatments against other pathogens (Manohar et al., 2018; Thiry et al., 2019; Tkhilaishvili et al., 2020), its application to *Campylobacter* remains limited. Existing studies employing this model for *Campylobacter* have focused primarily on characterizing virulence rather than assessing therapeutic interventions (Senior et al., 2011; Bojanić et al., 2020; Gomes et al., 2024). To date, no published studies have assessed bacteriophage efficacy against *Campylobacter* in the *G. mellonella* model, or in any other model that is not based on chicken. This highlights a methodological gap and supports the rationale for the present study.

Therefore, the aim of the present work was to evaluate the efficacy of a novel phage cocktail composed of four bacteriophages targeting *Campylobacter.* The *in vitro* lytic activity of both the cocktail and the four individual phages was investigated against antimicrobial-susceptible and -resistant strains of *C. jejuni*, *C. coli* and *C. lari.* Furthermore, their therapeutic potential was assessed *in vivo* using the *G. mellonella* model, both as a monotherapy and in combination with antibiotics, to explore the suitability of this invertebrate host model for early-stage evaluation of phage-based therapies against *Campylobacter*.

## 2 Materials and methods

### 2.1 *Campylobacter* strains: growth conditions and antimicrobial susceptibility

Thirteen *Campylobacter* strains from different sources were used in this study, including six *C. jejuni*, six *C. coli* and one *C. lari*, (Table 1). These strains, stored at −80 °C in Vegitone Infusion Broth (Merck Millipore, Darmstadt, Germany) with 20% glycerol, were growth on Columbia blood agar plates supplemented with 5% (v/v) defibrinated sheep blood (Oxoid, Hampshire, England) at 41.5 °C overnight under microaerobic conditions (5% O_2_, 10% CO_2_, and 85% N_2_) using an INVIVO2 400 hypoxia workstation (Ruskinn Technology Ltd., Bridgend, UK).

**Table 1:** Code, origin and antibiotic susceptibility of the 13 *Campylobacter* strains used in this study.

Species	Strain code	Origin	Antibiotic susceptibility	
			Ciprofloxacin	Erythromycin
*C. jejuni*	CJE061	Chicken feces	Resistant	Susceptible
	CJE063	Chicken feces	Resistant	Susceptible
	CJE065	Chicken feces	Susceptible	Susceptible
	CJE079	Chicken feces	Resistant	Resistant
	CJE084	Chicken skin	Resistant	Resistant
	CJE090	Chicken feces	Susceptible	Susceptible
*C. coli*	CCO007	Human	Resistant	Resistant
	CCO017	Human	Resistant	Resistant
	CCO039	Human	Resistant	Resistant
	CCO052	Chicken skin	Resistant	Susceptible
	CCO075	Chicken feces	Resistant	Susceptible
	CCO091	Chicken feces	Susceptible	Susceptible
*C. lari*	CLA005	Human	Resistant	Resistant

For the preparation of bacterial cultures, *Campylobacter* cells grown on Columbia agar supplemented with 5% sheep blood were harvested in Vegitone Infusion Broth (Vegitone, Merck Millipore). The bacterial suspensions were adjusted to an optical density (OD) at 600 nm wavelength (OD_600_) of 0.6, corresponding to approximately 10^9^ CFU per milliliter (CFU/mL), using a Genesys 6 spectrophotometer (Thermo Spectronic, USA).

The antimicrobial susceptibility of the *Campylobacter* strains was assessed by disk diffusion method using disks of 5 μg for the fluoroquinolone, ciprofloxacin (Oxoid), and of 15 μg for the macrolide, erythromycin (Oxoid). This procedure was conducted in accordance with the antimicrobial susceptibility testing guidelines of the European Committee on Antimicrobial Susceptibility Testing (EUCAST, 2024).

### 2.2 Bacteriophages propagation

The four *Campylobacter* specific phages used in this study (Table 2) were previously isolated from different origins and classified as group II campylophages (Nafarrate et al., 2021). The phages were propagated using the double agar overlay method. Briefly, 600 μL of *Campylobacter* bacterial culture supplemented with 1 mM CaCl₂ and 10 mM MgSO₄ were mixed with 4 mL of NZCYM broth (Condalab, Madrid, Spain) soft agar (0.7% agar) and overlaid on NZCYM hard agar plates (1.2% agar). The plates were incubated overnight at 42 °C under microaerobic conditions. Then, 6 mL of SM buffer (50 mM Tris–HCl, 100 mM NaCl, 8 mM MgSO_4_, 0.01% gelatine, pH 7.5) was added to the plates and incubated overnight at 4 °C with orbital shaking (250 rpm). For the assays of phages morphology and stability, the gelatine-free SM buffer (100 mM NaCl, 25 mM Tris–HCl pH 7.5, 8 mM MgSO_4_) was used to minimize impurities. The resulting phage suspension was treated with 10% chloroform and centrifuged at 16,500 × *g* for 15 min at 4 °C to remove cellular debris. The resulting supernatant was collected, and its sterility/purity was verified by culturing on Tryptone Soya Agar (TSA, Oxoid, Hampshire, England) medium. Phage titers were determined by spot assay, where 10 μL of serial dilutions were plated on NZCYM soft agar overlay plates and incubated overnight at 42 °C under microaerobic conditions.

**Table 2:** Code, origin, classification (class and group) and morphology parameters (head diameter and tail length) of the four *Campylobacter* bacteriophages used in this study.

Phage code	Class/group	Origin	Morphology parameters (nm)	
			Head Ø	Tail length
CAM165	C*audoviricetes*/II	Patient	94.01 ± 8.12	115.60 ± 6.88
CAM295	C*audoviricetes*/II	Chicken feces	86.46 ± 5.17	123.20 ± 4.22
CAM303	C*audoviricetes*/II	Chicken feces	86.14 ± 6.28	131.51 ± 8.83
CAM310	C*audoviricetes*/II	Chicken feces	92.51 ± 2.61	128.23 ± 5.61

Head Ø, head diameter.

### 2.3 Bacteriophages morphology and stability

The morphology of the four bacteriophages was assessed by transmission electron microscopy (TEM) analysis, performed at Electron Microscopy Service in Centro Nacional de Biotecnología (CNB, Madrid, Spain). Purified highly concentrated phage lysates of 10^9^ plaque-forming units (PFU) per milliliter (PFU/mL) were prepared. Phage particles were absorbed onto carbon-coated collodion 400 mesh nickel grids (Gilder) for 2 min and stained with 2% aqueous uranyl acetate (Electron Microscopy Sciences) for 1 min. Grids were visualized in a JEOL JEM 1400 Flash electron microscope (JEOL, Tokyo, Japan) operating at 100 kV. Micrographs were taken with a Gatan OneView digital camera (Gatan, California, USA) at various magnifications. Dimensions of phage particles, including head diameter and tail length, were determined on micrographs at 50 K × magnification, with Gatan Micrograph Digital Software (version 3.61.4719.0).

The thermal stability of the phages was evaluated over 120 h at 37 °C and pH 7.5. Briefly, 10 mL of phage suspensions at approximately 10^8^ PFU/mL were prepared in SM buffer at pH 7.5 and stored at 37 °C. After 10, 24, 72, and 120 h of storage aliquots were collected for phage enumeration. Serial dilutions were plated onto NZCYM soft agar overlay plates and incubated overnight at 42 °C under microaerobic conditions.

### 2.4 *In vitro* activity of the bacteriophages

The lytic spectra of individual phages and the phage cocktail containing the four phages were evaluated against 13 *Campylobacter* strains, including six *C. jejuni*, six *C. coli* and one *C. lari*. Ten microliters of each phage suspension and the phage cocktail at approximately 10^8^ PFU/mL were added to each bacterial host lawn present on double-layered agar plates. The plates were incubated overnight at 42 °C under microaerobic conditions, and the appearance of clear zones was observed. Bacterial susceptibility to phage infection was categorized as resistant, if no lysis occurred; moderately susceptible, if single plaques or opaque confluent lysis were observed; susceptible, if clear confluent lysis with a few isolated colonies was observed; and highly susceptible if clear confluent lysis was detected.

In addition, the phage reduction capacity against the *C. jejuni* CJE065 strain was evaluated in liquid medium, following the method described by Steffan et al. (2022) with some modifications. Briefly, a fresh bacterial culture was prepared in Vegitone broth and adjusted to a bacterial density of 10^5^ CFU/mL. Phage cocktails at 10^4^, 10^5^ and 10^6^ PFU/mL were prepared by combining equal volumes of each phage suspension, previously adjusted to the corresponding titers in Vegitone broth, to be applied at different multiplicity of infections (MOIs) levels of 0.1, 1 and 10, respectively. In particular, aliquots of 150 μL of bacterial suspension and 150 μL of phage solutions were added into individual wells of sterile flat bottom 96-well microplates (Nunc™ MicroWell™ with Nunclon Delta surface, Thermo-Scientific, Roskilde, Denmark). Vegitone broth was used as a negative control. Plates were incubated at 37 °C under microaerobic conditions with gentle shaking (365 cpm). Culture density was assessed by measuring OD_600_ every 20 min for 70 h using a plate reader (BioTek Synergy H1 Multimode Reader) equipped with a CO_2_ and O_2_ gas controller. Each condition was tested in triplicate per plate, and the experiments were performed in triplicate on different days.

### 2.5 *In vivo* activity of the bacteriophages

The *G. mellonella* host model was used to study the virulence of *Campylobacter* and the efficacy of different phage-treatments against *Campylobacter* infection, following the methodology describe previously with some modifications (Hernando-Ortiz et al., 2021). Larvae of *G. mellonella*, weighing between 0.2 and 0.5 g, were obtained from DNAT Ecosistemas (Toledo, Spain). One day before starting the experiments, the caterpillars were placed in groups of 20 in Petri dishes and maintained at 37 °C in the dark without food to adapt them to the experimental conditions. On the day of the experiment, the last left leg of each larva was cleaned with 70% ethanol and 10 μL of the different cell suspensions and/or treatments were inoculated with a precision syringe (Agilent, USA). In all trials, two uninfected controls were included: a group of untouched larvae and a group of larvae injected with 10 μL DPBS (Dulbecco’s Phosphate Buffered Saline, Sigma Aldrich, Missouri, USA) buffer to observe a possible impact of the injection. Larvae in Petri dishes were incubated at 37 °C in the dark for 120 h and larval survival was monitored every 24 h, removing the dead larvae melanized and/or without movement. Each trial was performed at least three times on different weeks.

#### 2.5.1 Virulence assessment of *Campylobacter*

The virulence of the 13 *Campylobacter* strains was evaluated in *G. mellonella in vivo* model. The bacterial suspensions at 10^9^ CFU/mL, prepared as described above, were washed twice in DPBS buffer via centrifugation at 8000 × *g* for 5 min at 4 °C. Inoculum concentrations were confirmed by colony counting on RAPID’*Campylobacter* agar (BioRad, France). After testing different concentrations (10^4^, 10^5^, 10^6^ and 10^7^ CFU/larva), the latter concentration was chosen to monitor the virulence of each *Campylobacter* strain. A total of 1,330 larvae were used to study the infection caused by different *Campylobacter* strains.

#### 2.5.2 Phage-treatments against *Campylobacter* infection

The efficacy of treatments using a four-phage cocktail and two antibiotics (ciprofloxacin and erythromycin) was evaluated *in vivo* against the *Campylobacter* infection. Groups of 20 larvae were infected with the *C. jejuni* CJE065 strain (10^7^ CFU/larva) and treated with the phage cocktail and two antibiotics, both as monotherapy and in combination. Monotherapy consisted in the application of the phage cocktail at 10^6^, 10^7^ and 10^8^ PFU/larva, at a MOI of 0.1, 1, and 10, respectively, or in the application of ciprofloxacin (0.5 μg/larva and 5 μg/larva) or erythromycin (1.5 μg/larva and 15 μg/larva). The combination of phage-antibiotic therapy involved the administration of the phage cocktail at a MOI of 10 together with ciprofloxacin (0.5 μg/larva and 5 μg/larva) or erythromycin (1.5 μg/larva and 15 μg/larva). In addition, the toxicity of the phages and antibiotics was assessed by testing uninfected larvae in the presence of the highest concentrations of each antimicrobial solution (phage cocktail at a MOI of 10, ciprofloxacin at 5 μg/larva, and erythromycin at 15 μg/larva), both as monotherapy and as combined treatment. A total of 1,965 larvae were utilized to evaluate treatments against *Campylobacter* infection.

### 2.6 Statistical analysis

Data preparation, visualization, and statistical analysis were conducted with R software v4.3.1 (R Core Team, 2023), GraphPad Prism 5 software (GraphPad Software, 2007, USA) DescTools package v0.99.57 (Signorell, 2023) and SPSS v24.0 (IBM Corp, 2016, USA). ComplexHeatmap package was used for Host range visualization. The analysis of *G. mellonella* survival was performed by the Kaplan–Meier method and differences between the survival rates were obtained and compared by the log-rank test. For all statistical analyses, values of *p* < 0.05 were considered statistically significant.

## 3 Results and discussion

*Campylobacter*-specific bacteriophages have emerged as a promising biocontrol strategy within the poultry production chain (Lavilla et al., 2023; Peh et al., 2023, 2024). Previous studies suggest their potential to reduce *Campylobacter* colonization in poulty and improve public health, with dosage and route of administration identified as key factors for treatment success (Janež and Loc-Carrillo, 2013; Kittler et al., 2013; Lavilla et al., 2023). In this context, the present study combines the *in vitro* evaluation of four lytic phages, applied individually and as a cocktail, with the pioneering use of the *Galleria mellonella* model to assess the *in vivo* efficacy of phage therapy, both as monotherapy and in combination with antibiotics, against this pathogen. The findings contribute to the development of new phage-based interventions to control *Campylobacter* across the farm-to-table continuum.

### 3.1 Morphology and stability of bacteriophages

**Figure 1:**
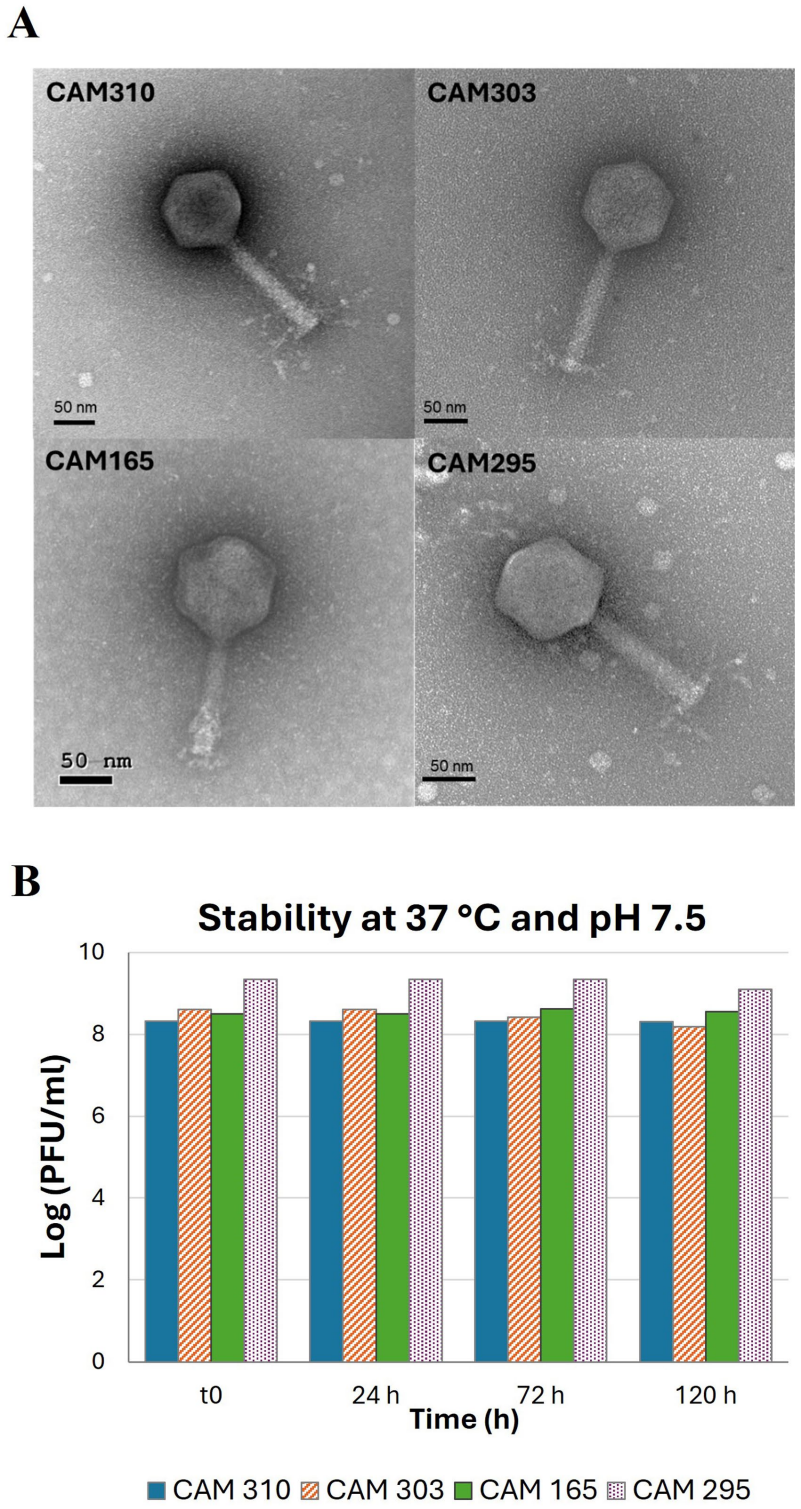
**(A)** Transmission electron microscopy images (scale bar = 50 nm) of the four bacteriophages used in this study: CAM310, CAM303, CAM165, and CAM295; and **(B)** their stability at 37 °C and pH 7.5 over 120 h.

The phages used in this study were characterized by TEM and the images (Figure 1A) revealed that they possess icosahedral heads with diameters ranging from 86 to 94 nm, and long, contractile tails of between 115 and 131 nm length (Table 2). The tails terminate in a basal plate with outwardly extending fibres. Based on the morphology, these phages were classified within the class *Caudoviricetes*, which encompasses the former family *Myoviridae* including phages with icosahedral heads and long contractile tails, such as the four characterized in the present work (Hwang et al., 2009; Steffan et al., 2022; Turner et al., 2023).

Overall, the four phages exhibited stability under the pH and temperature conditions tested. Specifically, titers remained above 8 log PFU/mL for 120 h at 37 °C in SM buffer at pH 7.5 (Figure 1B). The same conditions were previously tested for a *Campylobacter*-phage called CP6 and it was also stable although its stability was only assessed over a 60 min period (Zhang et al., 2024). The values obtained in the present study are also consistent with those of Ávila et al. (2023), who reported that *Clostridium tyrobutyricum*-phages remained stable for at least 7 days at 37 °C in SM buffer at pH 7.5. Furthermore, a neutral to slightly alkaline pH range (7.0–9.0) was identified as optimal for phage stability (Bagińska et al., 2024). In the present study, preliminary analyses were also performed with the four CAM phages at pH 9 and it was observed that their stability was preserved (data not shown).

### 3.2 Lytic potential of bacteriophages

**Figure 2:**
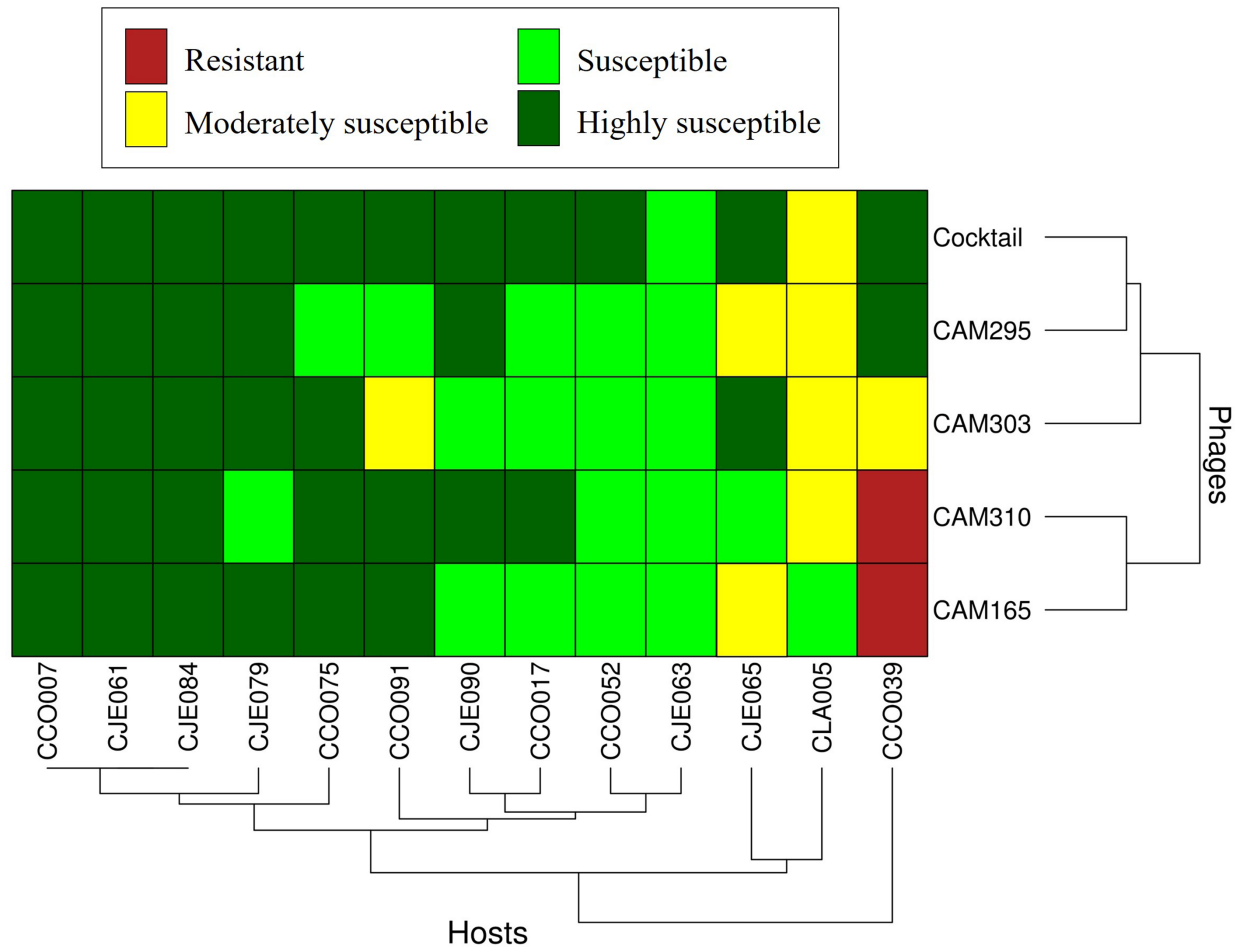
Host range of four *Campylobacter*-specific bacteriophages and a cocktail combining these four bacteriophages against 13 *Campylobacter* strains: 6 *C. jejuni*, 6 *C. coli*, and 1 *C. lari*. A color code was used to visualize bacterial susceptibility to phage infection: (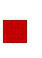) resistant (no lysis); (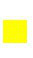) moderately susceptible (opaque confluent lysis); (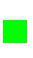) susceptible (clear confluent lysis with a few isolated colonies); (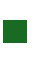) highly susceptible (clear confluent lysis).

The host range of each single phage and the phage cocktail containing these four phages is presented in Figure 2. All phages showed a broad lytic spectrum after individual application, lysing 92 to 100% of the strains tested. CAM310 and CAM165 were the only phages that were not able to lyse the *Campylobacter* CCO039 strain. However, this *Campylobacter* strain was highly and moderately susceptible to the phage CAM295 and CAM303, respectively, and highly susceptible to the phage cocktail. Overall, the phage cocktail showed an improved lytic spectrum and a broader host range. Of the *Campylobacter* strains, 85% (11 out of 13) showed high susceptibility to the phage cocktail. All these strains were highly susceptible to two or more phages separately, with the exception of the *C. coli* CCO052 strain, which was not susceptible to any single phage but showed susceptible to the cocktail. The remaining two *Campylobacter* strains (2 out of 13, 6%), the strains *C. lari* CLA005 and *C. jejuni* CJE063, were susceptible or moderately susceptible to the phage cocktail, maintaining the result observed with each phage.

These findings clearly indicate that phage cocktails broaden the host range and improve infection efficacy. In line with this, Steffan et al. (2022) demonstrated that the combination of *Campylobacter* phages from groups II and III enhanced the inhibition of this pathogen compared to the use of individual phages. On the one hand, Fischer et al. (2013) suggested that the use of phage cocktails might avoid the emergence of resistant *Campylobacter* mutants, as phages target multiple bacterial receptors. Beyond *Campylobacter*, phage cocktails have also been shown to be more effective than individual phages against other foodborne pathogens, including *Salmonella* in poultry (Torkashvand et al., 2024), *Pseudomonas fluorescens* in dairy products (Tayyarcan et al., 2024), and *Listeria monocytogenes* in seafood (Perera et al., 2015). Collectively, these findings support the ability of phage cocktails as an effective strategy for the biocontrol of foodborne pathogens across various sectors of the food industry.

### 3.3 *Campylobacter* virulence in *G. mellonella*

The *G. mellonella in vivo* model has been previously used to study the virulence of different foodborne bacterial pathogens including *Campylobacter* (Bojanić et al., 2020; Luiz de Freitas et al., 2021; Vinueza-Burgos et al., 2023; Gomes et al., 2024; Yan et al., 2025). This invertebrate model offers advantages in terms of economy, ethics and ease of handling, and is a promising alternative to vertebrate animals such as broiler chickens.

In the present study, different concentrations were tested and the inoculum of 1 × 10^7^ cells/larva, that were confirmed by culture (values between 7.9 × 10^8^ CFU/mL and 3.2 × 10^9^ CFU/mL), which were suitable to analyze the virulence of *Campylobacter*. This dose of infection was in line with that used in previous studies to assess *Campylobacter* virulence in the *G. mellonella* model (Senior et al., 2011; Bojanić et al., 2020).

**Figure 3:**
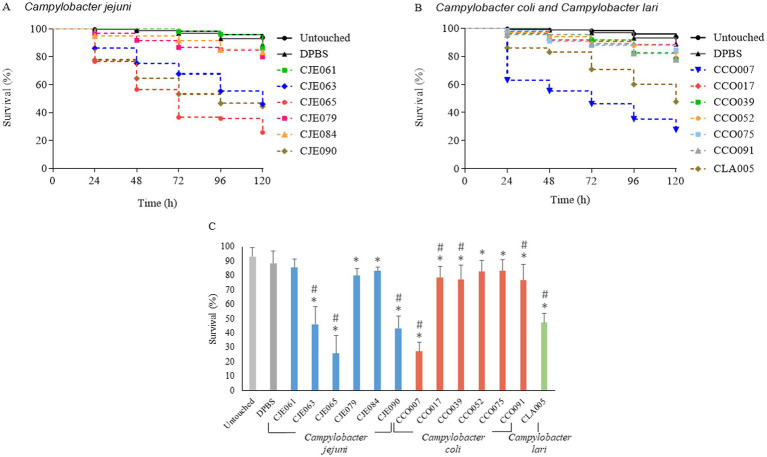
Survival curves of *G. mellonella* infected with 10^7^ CFU/larva of **(A)** *C. jejuni* strains and **(B)** *C. coli* and *C. lari* strains. **(C)** Survival percentages at 120 h post-infection. Statistically significant differences in pathogenicity of *Campylobacter* strains compared to two uninfected control, untouched larvae (*) and larvae injected with DPBS (#), calculated using the log-rank test (*p* < 0.05), are indicated. All detailed statistical analyses are available in the Supplementary Table S2.

The survival rates of untouched control larvae and DPBS-injected larvae were 93.8% ± 6 and 88.8% ± 8.4%, respectively, with no significant differences between them (*p* = 0.057) (Figure 3; Supplementary Tables S1, S2).

Analysis of 13 *Campylobacter* strains (six *C. jejuni*, six *C. coli*, and one *C. lari*) revealed strain-dependent virulence in *G. mellonella*, independent of species or isolation source, as was previously reported for *Campylobacter* (Champion et al., 2010; Bojanić et al., 2020; Gomes et al., 2024) and other foodborne pathogens (Liu et al., 2022; Vinueza-Burgos et al., 2023). Larval survival rates between 25.8 and 47.7% at 120 h post-infection were caused by the 38.5% of the strains (5 out of 13) from the three different species (CLA005, CJE063, CJE090, CCO007 and CJE065). In contrast, the remaining eight (8 out of 13, 61.5%) strains of both *C. jejuni* and *C. coli* species resulted in larval survival rates above 77.6% at 120 h (Supplementary Table S1). The strains CJE065 (Figure 3A) and CCO007 (Figure 3B) were identified as the most virulent, with survival rates of larvae at 120 h of only 25.8 and 27.3%, respectively, with no significant difference between them (*p* = 0.96). However, significant differences were observed between DPBS-injected larvae and those infected with these two strains and six more *Campylobacter* strains (CCO017, CCO039, CCO091, CLA005, CJE063, and CJE090) (*p* < 0.05) (Figure 3C).

Although only five of the 13 strains tested caused a marked reduction in *G. mellonella* survival, this result is consistent with previous studies reporting systematic differences in virulence among *Campylobacter* strains in both *G. mellonella* and vertebrate infection models (Champion et al., 2010; Bojanić et al., 2020; Gomes et al., 2024). Strain-specific virulence of *Campylobacter* in the *G. mellonella* model highlights the considerable genetic diversity within the genus. For example, Gomes et al. (2024) observed that virulence varied depending on the *C. coli* strain inoculated, and Senior et al. (2011) observed that *C. jejuni* virulence varied both between different multilocus sequence typing (MLST) groups and between strains within the same group. Furthermore, unlike what was observed in this study, differences in virulence between species have been previously reported, with *C. jejuni* being more virulent than *C. upsaliensis* and *C. helveticus* (Bojanić et al., 2020). Importantly, all strains included in this study were isolated from poultry or human sources, representing circulating isolates with heterogeneous pathogenic potential. Therefore, the observed variability is expected and reflects the well-documented genetic and phenotypic diversity within the *Campylobacter* genus. In this context, the *G. mellonella* model remains a useful platform for discriminating between highly virulent and low-virulence isolates and for selecting suitable strains for subsequent therapeutic evaluation.

Of the 13 *Campylobacter* strains tested in the present study, the strain CJE065 was selected for further assays in the *G. mellonella* model due to its high virulence in this model and susceptibility to ciprofloxacin and erythromycin (Table 1; Figure 3). This makes this strain a suitable candidate for evaluating the efficacy of combined phage–antibiotic treatments in *G. mellonella* model. Moreover, *C. jejuni* is considered a more significant food safety concern compared to *C. coli* (Andritsos et al., 2023; Ortiz et al., 2024) and its pathogenic potential could be linked to the presence of specific virulence genes, variations in surface structures, or differences in stress response mechanisms (Crofts et al., 2018). For example, proteins such as T6SS and CapC have been associated with increased *Campylobacter* virulence, as their alteration or inactivation significantly reduces mortality and pathogenic activity in *G. mellonella* larvae (Mehat et al., 2018; Liaw et al., 2019).

### 3.4 Efficacy of bacteriophages against *C. jejuni*

**Figure 4:**
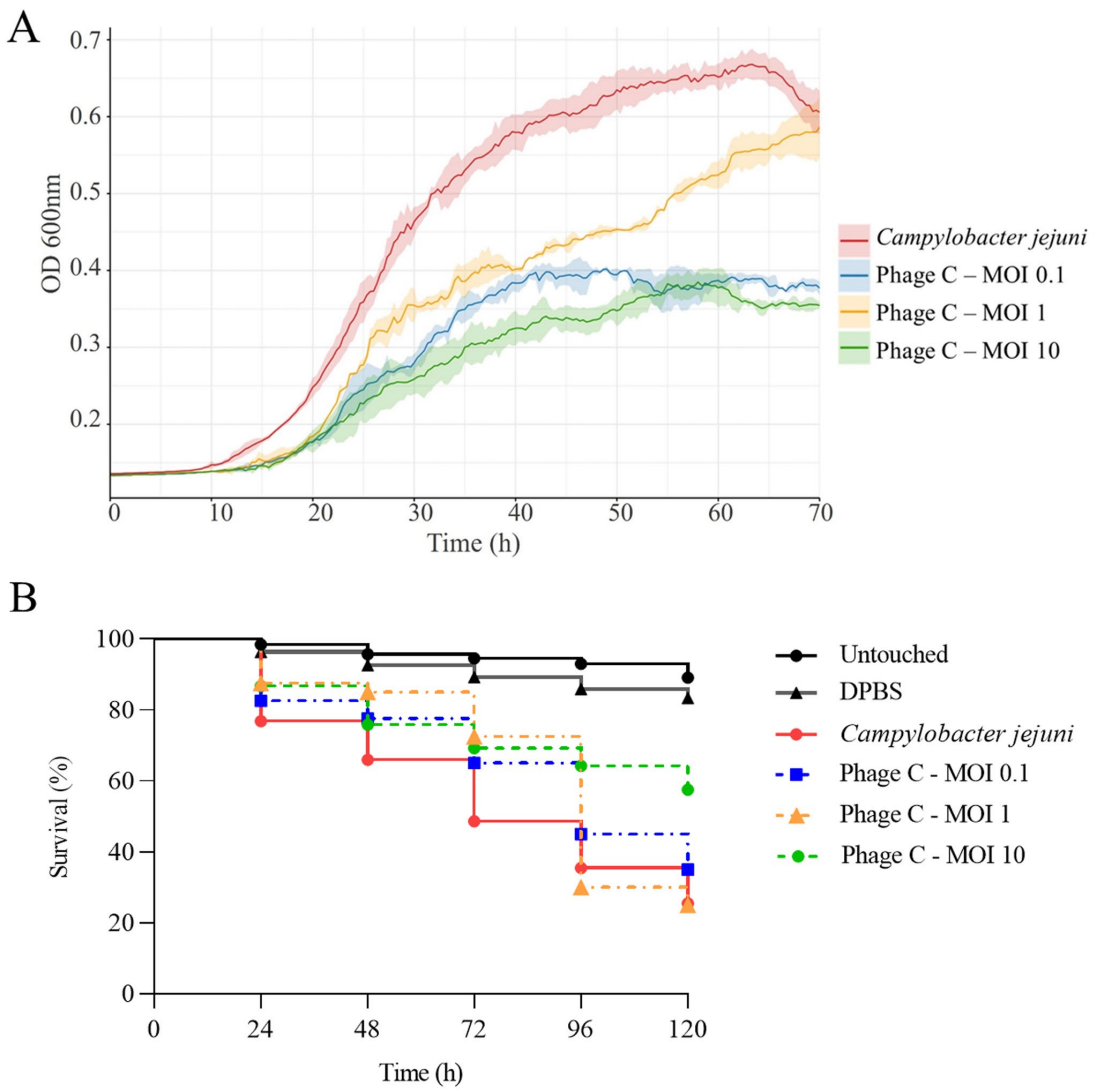
**(A)** Growth curves of the *C. jejuni* CJE065 strain in liquid medium alone and in the presence of the phage cocktail (Phage C) at different MOIs of 0.1, 1, and 10. Data represent mean OD600 values from three independent experiments, with standard deviations indicated by shaded areas around the mean. **(B)** Survival curves of *G. mellonella* infected with the *C. jejuni* CJE065 strain (10^7^ CFU/larva) and treated with the phage cocktail at MOIs of 0.1, 1, and 10. Untouched larvae and larvae injected with DPBS were included as a control.

The efficacy of the phage cocktail applied at MOIs of 0.1, 1 and 10, against *C. jejuni* was evaluated both *in vitro* and *in vivo* assays (Figure 4).

The *in vitro* assays showed that the phage cocktail at MOIs of 0.1 and 10 in Vegitone broth was able to inhibit the growth of *C. jejuni* compared to the control (Figure 4A). In presence of the phage cocktail at all MOIs tested, the lag phase of bacteria was prolonged by 7 h, and the bacterial growth rate was reduced by more than 50% in the first 50 h. This reduction was maintained at MOIs of 0.1 and 10. However, the phages at MOI of 1 initially delayed the growth, but regrowth was observed after 50 h. This non-linear pattern is consistent with established phage–host kinetic frameworks, where intermediate phage-to-bacterium ratios may not reach the inundation or proliferation thresholds needed to sustain long-term suppression (Cairns et al., 2009; Kasman et al., 2002). Under these conditions, it is plausible that the selective pressure exerted at an MOI of 1 favored the early expansion of partially resistant subpopulations, whereas higher or lower MOIs followed distinct density-dependent regimes that impeded regrowth.

The application of phage cocktails in liquid media has previously been reported to affect the growth of *Campylobacter* spp. For instance, Peh et al. (2023) demonstrated that several phage combinations at MOI of 10 prolonged the bacterial lag phase up to 9 h and reduced the growth rate for up to 24 h. Similar effects have been observed in other pathogens, the use of phage cocktails at MOI of 1 against *Vibrio cholerae* (Yen et al., 2017) and at MOIs ranging from 0.1 to 100 against *Salmonella enterica* (Islam et al., 2019) inhibited bacterial growth for up to 12 h.

The bacteriophage application also showed promising results in the treatment against the *Campylobacter* infection caused in the *G. mellonella* model (Figure 4B). All four phages used in the phage cocktail maintained *in vitro* stability over 120 h duration of the *in vivo G. mellonella* assays. In this infection model the phage cocktail at MOI of 10 showed the highest efficacy against *Campylobacter* infection. Notably, the survival of *G. mellonella* larvae increased significantly from 25.5% in untreated infected controls to 57.5% in infected larvae treated at this highest MOI, with significant differences between them (*p* < 0.001). A temporary increase in larval survival was observed in the groups treated with MOIs of 1 and 0.1, reaching 72.5 and 65.0% respectively, at 72 h, compared to 48.6% in the infected controls. However, statistically significant differences were only observed between the MOI 1 group and the infected control (*p* = 0.007). However, this difference was not maintained until 120 h, at which point the survival rates in both treated groups were similar to those of infected controls.

The *G. mellonella* model has already been successfully employed to study phage applications against several bacterial pathogens, such as methicillin-resistant *Staphylococcus aureus* (MRSA) (Tkhilaishvili et al., 2020), *Shigella flexneri* (Filippov et al., 2022), or *S. enterica* (Nale et al., 2021; Kosznik-Kwaśnicka et al., 2022). However, to the best of our knowledge, research utilizing the *G. mellonella* infection model for *Campylobacter* studies is limited, and none of these have examined the effectiveness of phage against this pathogen.

Bacteriophages against *Campylobacter* have been examined in poultry trials to reduce the *in vivo* pathogen load with minimal disruption to both the host’s natural microbiota and the environment (Lavilla et al., 2023). This strategy emerged to mitigate *Campylobacter* contamination in poultry production at farm level, achieved significant reduction of 1 to 2-log CFU/g in commercial poultry food products (D'Angelantonio et al., 2021; Peh et al., 2023). It is worth highlighting that Koutsoumanis et al. (2020) estimates that a 3-log reduction in pre-slaughter *Campylobacter* intestinal load would reduce the risk of human campylobacteriosis associated with poultry meat consumption by 58%. This promising strategy requires further research to identify effective and potent *Campylobacter*-specific bacteriophages with high potential as biocontrol agents. In this context, an invertebrate model such as *G. mellonella* offers a particularly valuable intermediate step for validating the *in vivo* efficacy of phages and optimizing key parameters, such as the MOI, before progressing to more complex vertebrate models, as recently reported (Barton et al., 2024). Our findings further support the usefulness of this approach for studying phages against *Campylobacter*. Nevertheless, a limitation of the present study is the absence of additional strains from other *Campylobacter* species. Future work will extend the *in vivo* assessment to include other species, such as *C. coli* and *C. lari*, to determine whether the observed phage–antibiotic interactions for *C. jejuni* CJE065 are conserved across species. The utilization of diverse *Campylobacter* strains will enable the evaluation of strain- and species-level variability in the *G. mellonella* model. Taken together, the data reveal a biologically coherent MOI-dependent pattern, in which differences between low, intermediate and high phage-to-bacterium ratios can lead to clearance, transient inhibition or regrowth, consistent with density-dependent threshold dynamics previously characterized in phage–*Campylobacter* systems and other host–phage models (Cairns et al., 2009; Kasman et al., 2002).

### 3.5 Efficacy of bacteriophage and antibiotic therapies during *G. mellonella* infection with *C. jejuni*

The combined therapy of bacteriophages and antibiotics has emerged as a promising strategy to combat bacterial infections, particularly in the context of rising antimicrobial resistance. Previous studies have demonstrated that this synergy not only enhances bactericidal efficacy but also reduces the *in vitro* resistance emergence (Li et al., 2021) and has also shown *in vivo* success in treating MRSA infection in *G. mellonella* model (Loganathan et al., 2024). In fact, *G. mellonella* invertebrate model has allowed evaluating the effectiveness of different treatments and optimal doses to be assessed prior to their application in poultry production systems (Lacharme-Lora et al., 2019; Nale et al., 2021).

The present study evaluated the toxicity and effect of the most effective concentration of phage cocktail (MOI of 10) in monotherapy and in combination with the antibiotics ciprofloxacin and erythromycin, for treating the infection caused by the *C. jejuni* CJE065 strain in the *G. mellonella* model. The two control larval groups showed survival rates of 89.1% ± 9.4% for untouched larvae and 83.3% ± 12.2% for larvae injected with DPBS at 120 h post-injection, with no significant differences between them (*p* = 0.052).

Toxicity testing of *G. mellonella* injected with phage-based and antibiotics-based treatments at the highest applied concentration, showed larval survival rates between 76.7 and 95% at 120 h (Supplementary Figure S1 and Supplementary Table S3). A slight decline in *G. mellonella* survival was observed after the phage cocktail administration. This may be attributed to the presence of toxic molecules, such as endotoxins, in the phage suspension, which cannot be completely eliminated during the phage purification process, as was previously reported (Filippov et al., 2022).

**Figure 5:**
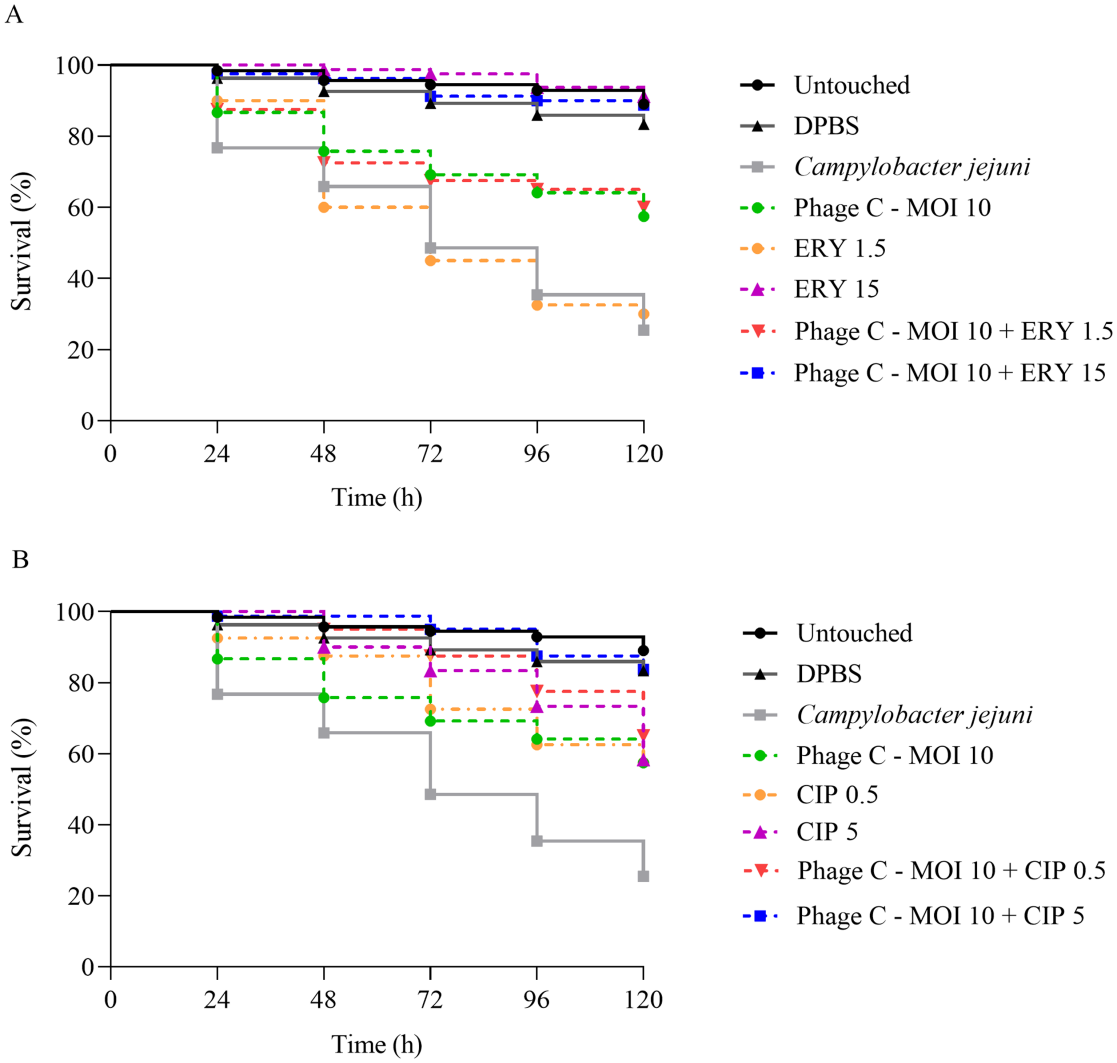
Effect of phage-based, antibiotic-based, and combined phage plus antibiotic-based treatments on the survival of *Campylobacter*-infected *G. mellonella*. Survival curves of *G. mellonella* infected with the *C. jejuni* CJE065 strain (10^7^ CFU/larva) in the presence of: **(A)** The antibiotic erythromycin (ERY) at dosages of 1.5 μg/larva and 15 μg/larva, in monotherapy and in combination with the phage cocktail (Phage C) at MOI 10; and **(B)** the antibiotic ciprofloxacin (CIP) at dosages of 0.5 μg/larva and 5 μg/larva, in monotherapy and in combination with the phage cocktail at MOI 10. All detailed statistical analyses are available in the Supplementary Table S4.

The antibiotic treatment with erythromycin at 15 μg/larva was the most effective, increasing survival from 25.5 to 91.3% after 120 h (*p* < 0.001) (Figure 5A and Supplementary Table S4). In contrast, treatment with erythromycin at 1.5 μg/larva showed no significant differences compared to the infected and untreated group. The combination of the phage cocktail with this antibiotic was significantly effective under all conditions tested. The combination with erythromycin at 15 μg/larva significantly increased the larvae survival by 63.3% over the untreated control (88.8% survival rate at 120 h). However, no differences were observed between the phage cocktail application in monotherapy and in combination with erythromycin at 1.5 μg/larva. In the case of the ciprofloxacin antibiotic, the concentration of 5 μg/larva combined with phage cocktail was the most active, increasing larvae survival from 25.5% without treatment to 83.8% (*p* < 0.001) after 120 h. This concentration in monotherapy significantly achieved a larvae survival of 58.3% (*p* < 0.001) compared with larvae infected and untreated (*p* < 0.001) (Figure 5B and Supplementary Table S4). The treatment of ciprofloxacin at 0.5 μg/larva, both in monotherapy and with phages combination, enhanced survival to 57.5 and 65%, respectively. These results showed significant differences compared to the infected and untreated larvae (*p* < 0.001 for both).

Most *in vitro* studies have analyzed the synergistic activity of phages with antibiotics against pathogens, showing that phages generally reduce the minimum inhibitory concentration of the antibiotic (Moryl et al., 2025). The combination of staphylococcal phage with oxacillin was more effective in eradicating *S. aureus*, including MRSA clinical isolates, than either treatment alone, both *in vitro* and in the *G. mellonella* animal model (Simon et al., 2021; Loganathan et al., 2024). The combination of phages and antibiotics in the *G. mellonella* model was also tested against infections caused by multidrug-resistant *Klebsiella* strains, and was reported to be safe and highly effective (Kelly and Jameson, 2024). In the case of *Campylobacter*, a previous *in vitro* study combining two lysogenic campylophages, vB_CjeMPC10 and vB_CjeM-PC22, with ethylenediaminetetraacetic acid (EDTA) showed reduced bacterial counts at 42 °C and inhibition of resistant bacterial regrowth for up to 48 h of exposure (Lwin et al., 2024). However, the effectiveness of combining phages and antibiotics against *Campylobacter* has not yet been evaluated *in vitro* and *in vivo*.

Bacteriophages offer multiple advantages, such as high specificity to target bacteria and safety for animals and humans, with no adverse effects observed in poultry (Chung et al., 2023; D'Angelantonio et al., 2021). The application of bacteriophages represents a promising alternative to reducing antibiotic use in poultry farming, thereby contributing to the management of antibiotic resistance, and mitigating the negative impacts associated with traditional chemical disinfectants (Jordá et al., 2023). Moreover, combining phages and antibiotics can reduce resistance levels and constitutes a safe treatment for susceptible and resistant strains, as it involves different targets and mechanisms of action that might enhance the antibiotic’s therapeutic effect (Morrisette et al., 2020; Li et al., 2021; Simon et al., 2021). Additionally, this phage approach is widely supported, with 90% of producers and 85% of consumers in favor of their use as a biocontrol method in poultry farms (Lavilla et al., 2023).

Collectively, the findings of the present study emphasize the promise of phage therapy as an effective strategy for controlling *Campylobacter*, both as a monotherapy and in combination with antibiotics. Furthermore, the use of *G. mellonella* enabled optimization of therapeutic dose selection and combinations, as well as rapid, ethical, and efficient toxicity assessment, thereby minimizing the use of vertebrate animals. These results underscore the relevance of employing this model as an essential intermediate step before advancing to trials in chickens.

## 4 Conclusion

The increasing incidence of campylobacteriosis, the alarming emergence and dissemination of multidrug-resistant *Campylobacter* strains, and the high prevalence of this zoonotic pathogen on farms underscore the urgent need for innovative and effective control strategies. In this study, four previously isolated C*ampylobacter*-specific phages were evaluated for their potential as biocontrol agents. Phages were shown to be stable under the pH and temperature conditions used in the *in vivo* assays. The phage cocktail combining the four phages exhibited an improved and broader lytic spectrum compared to individual phages, successfully infecting all tested *Campylobacter* strains.

The application of the phage cocktail, both alone and in combination with ciprofloxacin and erythromycin, significantly enhanced the survival of infected *G. mellonella* larvae, supporting its therapeutic potential against *C. jejuni in vivo*. The use of this invertebrate model offers a valuable platform for the rational and safe development of novel phage-based biocontrol strategies for the poultry industry.

These results not only reinforce the potential of phage cocktails as effective biocontrol tools for controlling *Campylobacter* in the food chain, but also highlight the relevance of *G. mellonella* as a simple, ethical and scalable infection model for early-stage evaluation of phage therapies. Although the results of both *in vitro* and *in vivo* studies are encouraging, further research is required to optimize phage application protocols including dosing regimen, frequency and routes of administration, and to assess phage stability and efficacy under variable farm conditions, such as temperature fluctuations, humidity, and the presence of organic matter in complex poultry environments.

Moreover, future studies will expand the *in vivo* evaluation to include additional strains of other *Campylobacter* species, such as *C. jejuni*, *C. coli*, and *C. lari*, thereby enhancing the comprehension of the outcomes of this study and their potential applications.

## Data Availability

The original contributions presented in the study are included in the article/Supplementary material, further inquiries can be directed to the corresponding author.
