# Supplementary material for: Broad-spectrum phage cocktail targeting Campylobacter improves survival in Galleria mellonella, a bridging host model for poultry biocontrol
Source: Front Microbiol. 2026 Apr 15;17:1744469. doi: 10.3389/fmicb.2026.1744469 (PMC13125106; doi:10.3389/fmicb.2026.1744469)
Supplement: Supplementary file 1 [file Supplementary_file_1.docx]

**Supplementary Material**

**Table S1.** Survival percentage of *G. mellonella* larvae infected with six *C. jejuni* and *C. coli* strains and one *C. lari* strain over 120 h.

| **Species/conditions** | **Survival percentage** | | | | | | | | | | | | | |
| --- | --- | --- | --- | --- | --- | --- | --- | --- | --- | --- | --- | --- | --- | --- |
|  | **24 h** | |  | **48 h** | |  | **72 h** | |  | **96 h** | |  | **120 h** | |
|  | **%** | **SD** |  | **%** | **SD** |  | **%** | **SD** |  | **%** | **SD** |  | **%** | **SD** |
| Untouched | 99.6 | 1.3 |  | 98.7 | 2.3 |  | 98.2 | 3.0 |  | 96.0 | 4.9 |  | 93.8 | 6.0 |
| DPBS | 99.6 | 1.2 |  | 98.8 | 2.0 |  | 96.7 | 4.2 |  | 92.9 | 5.9 |  | 88.8 | 8.4 |
| *Campylobacter coli* CCO007 | 63.1 | 8.7 |  | 55.4 | 14.0 |  | 46.2 | 13.2 |  | 35.4 | 5.0 |  | 27.7 | 6.4 |
| *Campylobacter coli* CCO017 | 97.6 | 2.9 |  | 91.8 | 7.7 |  | 90.6 | 6.1 |  | 88.2 | 9.0 |  | 78.8 | 7.7 |
| *Campylobacter coli* CCO039 | 96.5 | 4.8 |  | 94.1 | 7.5 |  | 91.8 | 5.1 |  | 82.4 | 5.7 |  | 77.6 | 7.7 |
| *Campylobacter coli* CCO052 | 96.9 | 2.9 |  | 93.8 | 3.2 |  | 89.2 | 7.9 |  | 87.7 | 6.4 |  | 83.1 | 7.5 |
| *Campylobacter coli* CCO075 | 98.5 | 2.9 |  | 90.8 | 10.0 |  | 87.7 | 8.2 |  | 87.7 | 8.2 |  | 84.6 | 11.0 |
| *Campylobacter coli* CCO091 | 95.6 | 7.1 |  | 95.6 | 7.1 |  | 88.9 | 11.3 |  | 82.2 | 15.6 |  | 77.8 | 9.9 |
| *Campylobacter lari* CLA005 | 86.2 | 13.7 |  | 83.1 | 19.4 |  | 70.8 | 14.5 |  | 60.0 | 9.0 |  | 47.7 | 6.4 |
| *Campylobacter jejuni* CJE061 | 100.0 | 0.0 |  | 100.0 | 0.0 |  | 98.0 | 2.8 |  | 96.0 | 5.7 |  | 86.0 | 8.5 |
| *Campylobacter jejuni* CJE063 | 86.2 | 13.6 |  | 75.4 | 5.0 |  | 67.7 | 3.5 |  | 55.4 | 10.0 |  | 46.2 | 12.7 |
| *Campylobacter jejuni* CJE065 | 76.7 | 19.9 |  | 56.7 | 17.5 |  | 36.7 | 9.4 |  | 35.8 | 6.6 |  | 25.8 | 5.8 |
| *Campylobacter jejuni* CJE079 | 96.7 | 5.8 |  | 91.7 | 10.4 |  | 86.7 | 10.4 |  | 85.0 | 8.7 |  | 80.0 | 5.0 |
| *Campylobacter jejuni* CJE084 | 95.0 | 8.7 |  | 95.0 | 8.7 |  | 91.7 | 5.8 |  | 85.0 | 0.0 |  | 83.3 | 2.9 |
| *Campylobacter jejuni* CJE090 | 77.8 | 16.3 |  | 64.4 | 24.7 |  | 53.3 | 17.0 |  | 46.7 | 14.8 |  | 44.4 | 12.0 |

h: hours; SD: standard deviation; %: percentage.

**Table S2.** Statistical analysis of differences in virulence of six *C. jejuni* and *C. coli* strains and one *C. lari* strain in *G. mellonella* model infection. The analysis was performed using the log-rank test and values of p <0.05 were considered statistically significant.

| **Condition** |  |  | **p-values** | | | | | | | | | | | | | | | |
| --- | --- | --- | --- | --- | --- | --- | --- | --- | --- | --- | --- | --- | --- | --- | --- | --- | --- | --- |
|  | Untouched  larvae | | DPBS^*^ | *Campylobacter jejuni* | | | | | |  | *Campylobacter coli* | | | | | |  | *Campylobacter*  *lari*  CLA005 |
|  |  | |  | CJE061 | CJE063 | CJE065 | CJE075 | CJE084 | CJE090 |  | CCO007 | CCO017 | CCO039 | CCO052 | CCO075 | CCO091 |  |  |
| Untouched larvae |  | | 0.057 | 0.069 | <0.001 | <0.001 | 0.001 | 0.007 | <0.001 |  | <0.001 | <0.001 | <0.001 | 0.005 | 0.014 | <0.001 |  | <0.001 |
| DPBS* | 0.057 | |  | 0.634 | <0.001 | <0.001 | 0.052 | 0.215 | <0.001 |  | <0.001 | 0.020 | 0.009 | 0.185 | 0.304 | 0.033 |  | <0.001 |
| CJE061 | 0.069 | | 0.634 |  | <0.001 | <0.001 | 0.336 | 0.610 | <0.001 |  | <0.001 | 0.263 | 0.193 | 0.585 | 0.741 | 0.239 |  | <0.001 |
| CJE063 | <0.001 | | <0.001 | <0.001 |  | 0.003 | <0.001 | <0.001 | 0.557 |  | 0.010 | <0.001 | <0.001 | <0.001 | <0.001 | 0.001 |  | 0.744 |
| CJE065 | <0.001 | | <0.001 | <0.001 | 0.003 |  | <0.001 | <0.001 | 0.060 |  | 0.960 | <0.001 | <0.001 | <0.001 | <0.001 | <0.001 |  | 0.001 |
| CJE079 | 0.001 | | 0.052 | 0.336 | <0.001 | <0.001 |  | 0.633 | <0.001 |  | <0.001 | 0.921 | 0.786 | 0.652 | 0.512 | 0.814 |  | <0.001 |
| CJE084 | 0.007 | | 0.215 | 0.610 | <0.001 | <0.001 | 0.633 |  | <0.001 |  | <0.001 | 0.553 | 0.431 | 0.969 | 0.864 | 0.488 |  | <0.001 |
| CJE090 | <0.001 | | <0.001 | <0.001 | 0.557 | 0.060 | <0.001 | <0.001 |  |  | 0.095 | <0.001 | <0.001 | <0.001 | <0.001 | <0.001 |  | 0.378 |
| CCO007 | <0.001 | | <0.001 | <0.001 | 0.010 | 0.960 | <0.001 | <0.001 | 0.095 |  |  | <0.001 | <0.001 | <0.001 | <0.001 | <0.001 |  | 0.004 |
| CCO017 | <0.001 | | 0.020 | 0.263 | <0.001 | <0.001 | 0.921 | 0.553 | <0.001 |  | <0.001 |  | 0.819 | 0.552 | 0.408 | 0.846 |  | <0.001 |
| CCO039 | <0.001 | | 0.009 | 0.193 | <0.001 | <0.001 | 0.786 | 0.431 | <0.001 |  | <0.001 | 0.819 |  | 0.441 | 0.326 | 0.990 |  | <0.001 |
| CCO052 | 0.005 | | 0.185 | 0.585 | <0.001 | <0.001 | 0.652 | 0.969 | <0.001 |  | <0.001 | 0.552 | 0.441 |  | 0.831 | 0.506 |  | <0.001 |
| CCO075 | 0.014 | | 0.304 | 0.741 | <0.001 | <0.001 | 0.512 | 0.864 | <0.001 |  | <0.001 | 0.408 | 0.326 | 0.831 |  | 0.394 |  | <0.001 |
| CCO091 | <0.001 | | 0.033 | 0.239 | 0.001 | <0.001 | 0.814 | 0.488 | <0.001 |  | <0.001 | 0.846 | 0.990 | 0.506 | 0.394 |  |  | 0.002 |
| CLA005 | <0.001 | | <0.001 | <0.001 | 0.744 | 0.001 | <0.001 | <0.001 | 0.378 |  | 0.004 | <0.001 | <0.001 | <0.001 | <0.001 | 0.002 |  |  |
| DPBS*: Uninfected larvae injected with DPBS. | | | | | | | | | | | | | | | | | | |

**Table S3.** Statistical analysis of differences in toxicity of the phage cocktail at MOI 10, ciprofloxacin, erythromycin and their combinations in *G. mellonella* animal model. The analysis was performed using the log-rank test and values of p < 0.05 were considered statistically significant

| **Condition** | **p-values** | | | | | | |
| --- | --- | --- | --- | --- | --- | --- | --- |
|  | Untouched  larvae | DPBS* | Phage MOI 10 | Ciprofloxacin 5 µg/larva | Erythromycin  15 µg/larva | Phage MOI 10 + Ciprofloxacin  5 µg/larva | Phage MOI 10 + Erythromycin  15 µg/larva |
|  |  |  |  |  |  |  |  |
| Untouched larvae |  | 0.052 | <0.001 | 0.220 | 0.251 | 0.473 | 0.523 |
| DPBS* | 0.052 |  | 0.116 | 0.020 | 0.060 | 0.124 | 0.146 |
| MOI 10 | <0.001 | 0.116 |  | <0.001 | 0.012 | 0.026 | 0.033 |
| Ciprofloxacin 5 µg/larva | 0.220 | 0.020 | <0.001 |  | 0.790 | 0.833 | 0.779 |
| Erythromycin 15 µg/larva | 0.251 | 0.060 | 0.012 | 0.790 |  | 0.666 | 0.643 |
| MOI 10 + Ciprofloxacin 5 µg/larva | 0.473 | 0.124 | 0.026 | 0.833 | 0.666 |  | 0.966 |
| MOI 10 + Erythromycin 15 µg/larva | 0.523 | 0.146 | 0.033 | 0.779 | 0.643 | 0.966 |  |
| DPBS*: Uninfected larvae injected with DPBS. | | | | | | | |

**Table S4.** Statistical analysis of survival curves of *G. mellonella* infected with the *C. jejuni* CJE065 strain and treated with the bacteriophages cocktail (MOI 0.1, 1 and 10) and with the antibiotics, ciprofloxacin (0.5 µg/larva and 5 µg/larva) and erythromycin (1.5 µg/larva and 15 µg/larva). *G. mellonella* larvae were infected with 10^7^ CFU/larva and the treatments were performed in monotherapy and in combination. The analysis was performed using the log-rank test and values of p < 0.05 were considered statistically significant.

|  | **p-values** | | | | | | | | | | | | | | | | | | | |
| --- | --- | --- | --- | --- | --- | --- | --- | --- | --- | --- | --- | --- | --- | --- | --- | --- | --- | --- | --- | --- |
| **Treatment** | Untouched  larvae | DPBS* | *C. jejuni* CJE065 infected and untreated | **Monotherapy** | | | | | | | | |  | **Combination therapy** | | | | | | |
|  |  |  |  | Phage MOI | | |  | Ciprofloxacin | |  | Erythromycin | |  | Phage MOI 10 + | | | | | | |
|  |  |  |  |  |  |  |  |  |  |  |  |  |  | Ciprofloxacin | | |  | Erythromycin | | |
|  |  |  |  | 0.1 | 1 | 10 |  | 0.5  µg/larva | 5  µg/larva |  | 1.5  µg/larva | 15  µg/larva | |  | 0.5  µg/larva | 5  µg/larva | |  | 1.5  µg/larva | 15  µg/larva |
| Untouched larvae |  | 0.052 | <0.001 | <0.001 | <0.001 | <0.001 |  | <0.001 | <0.001 |  | <0.001 | 0.559 | |  | <0.001 | 0.219 | |  | <0.001 | 0.917 |
| DPBS* | 0.052 |  | <0.001 | <0.001 | <0.001 | <0.001 |  | <0.001 | <0.001 |  | <0.001 | 0.074 | |  | 0.010 | 0.840 | |  | <0.001 | 0.244 |
| *C. jejuni* CJE065 infected and untreated | <0.001 | <0.001 |  | 0.134 | 0.375 | <0.001 |  | <0.001 | <0.001 |  | 0.782 | <0.001 | |  | <0.001 | <0.001 | |  | <0.001 | <0.001 |
| MOI 0.1 | <0.001 | <0.001 | 0.134 |  | 0.605 | 0.027 |  | 0.057 | 0.007 |  | 0.363 | <0.001 | |  | 0.003 | <0.001 | |  | 0.062 | <0.001 |
| MOI 1 | <0.001 | <0.001 | 0.375 | 0.605 |  | 0.004 |  | 0.015 | <0.001 |  | 0.463 | <0.001 | |  | <0.001 | <0.001 | |  | 0.019 | <0.001 |
| MOI 10 | <0.001 | <0.001 | <0.001 | 0.027 | 0.004 |  |  | 0.857 | 0.538 |  | 0.002 | <0.001 | |  | 0.233 | <0.001 | |  | 0.836 | <0.001 |
| Ciprofloxacin 0.5 µg/larva | <0.001 | <0.001 | <0.001 | 0.057 | 0.015 | 0.857 |  |  | 0.696 |  | 0.007 | <0.001 | |  | 0.342 | 0.001 | |  | 0.989 | <0.001 |
| Ciprofloxacin 5 µg/larva | <0.001 | <0.001 | <0.001 | 0.007 | <0.001 | 0.538 |  | 0.696 |  |  | <0.001 | <0.001 | |  | 0.489 | 0.001 | |  | 0.747 | <0.001 |
| Erythromycin 1.5 µg/larva | <0.001 | <0.001 | 0.782 | 0.363 | 0.463 | 0.002 |  | 0.007 | <0.001 |  |  | <0.001 | |  | <0.001 | <0.001 | |  | 0.015 | <0.001 |
| Erythromycin 15 µg/larva | 0.559 | 0.074 | <0.001 | <0.001 | <0.001 | <0.001 |  | <0.001 | <0.001 |  | <0.001 |  | |  | <0.001 | 0.152 | |  | <0.001 | 0.565 |
| MOI 10 + Ciprofloxacin 0.5 µg/larva | <0.001 | 0.010 | <0.001 | 0.003 | <0.001 | 0.233 |  | 0.342 | 0.489 |  | <0.001 | <0.001 | |  |  | 0.020 | |  | 0.393 | 0.003 |
| MOI 10 + Ciprofloxacin 5 µg/larva | 0.219 | 0.840 | <0.001 | <0.001 | <0.001 | <0.001 |  | 0.001 | 0.001 |  | <0.001 | 0.152 | |  | 0.020 |  | |  | 0.001 | 0.408 |
| MOI 10 + Erythromycin 1.5 µg/larva | <0.001 | <0.001 | <0.001 | 0.062 | 0.019 | 0.836 |  | 0.989 | 0.747 |  | 0.015 | <0.001 | |  | 0.393 | 0.001 | |  |  | <0.001 |
| MOI 10 + Erythromycin 15 µg/larva | 0.917 | 0.244 | <0.001 | <0.001 | <0.001 | <0.001 |  | <0.001 | <0.001 |  | <0.001 | 0.565 | |  | 0.003 | 0.408 | |  | <0.001 |  |
| DPBS*: Uninfected larvae injected with DPBS | | | | | | | | | | | | | | | | | | | | |

**Figure S1.** Survival curves of *G. mellonella* in the presence of the highest dose of the phage cocktail (Phage C - MOI 10) and antibiotics in monotherapy, ciprofloxacin 5 µg/larva (CIP 5) and erythromycin 15 µg/larva (ERY15) and in combination.

**
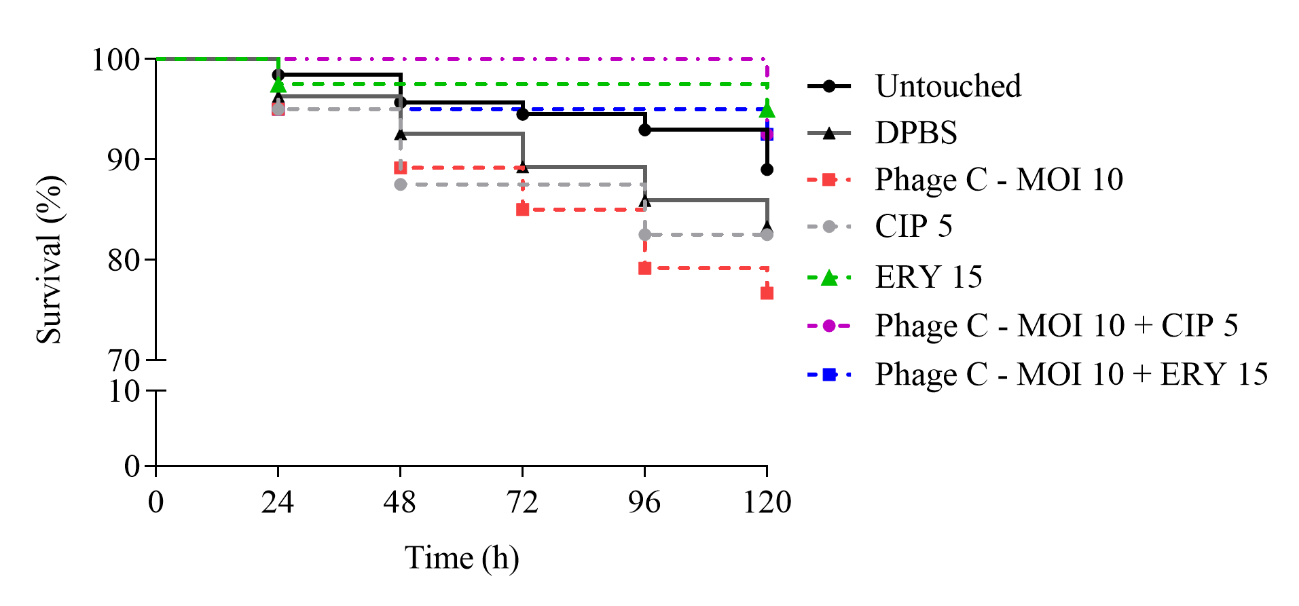
**
